# Antimicrobial Effect of a Peptide Containing Novel Oral Spray on *Streptococcus mutans*

**DOI:** 10.1155/2020/6853652

**Published:** 2020-03-10

**Authors:** Kaixin Xiong, Xuan Chen, Hantao Hu, Huihui Hou, Peng Gao, Ling Zou

**Affiliations:** ^1^State Key Laboratory of Oral Diseases, National Clinical Research Center for Oral Diseases, Sichuan University, Chengdu 610041, China; ^2^State Key Laboratory of Oral Diseases, National Clinical Research Center for Oral Diseases, Department of Conservation Dentistry and Endodontics, West China Hospital of Stomatology, Sichuan University, Chengdu 610041, China

## Abstract

**Objective:**

To investigate the antibacterial effect of a novel antimicrobial peptide containing oral spray GERM CLEAN on *Streptococcus mutans (S. mutans) in vitro* and further explore the related mechanisms at phenotypic and transcriptional levels.

**Methods:**

The disk diffusion method was used to preliminarily appraise the antimicrobial effect of GERM CLEAN. The minimal inhibitory concentration (MIC) of GREM CLEAN towards *S. mutans* was determined by the broth dilution method. *S. mutans* was determined by the broth dilution method.

**Results:**

The diameter (10.18 ± 1.744 mm) of inhibition zones formed by GERM CLEAN preliminarily indicated its inhibitory effect on the major cariogenic bacteria *S. mutans* was determined by the broth dilution method. *S. mutans* was determined by the broth dilution method. *S. mutans* was determined by the broth dilution method. *S. mutans* was determined by the broth dilution method. *gtfB*, *gtfC*, *gtfD*, and *ldh* were significantly repressed by treating with GERM CLEAN, and this was consistent with our phenotypic results.

**Conclusion:**

The novel antimicrobial peptide containing oral spray GERM CLEAN has an anti-*Streptococcus mutans* effect and the inhibitory property may be due to suppression of the virulence factors of *S. mutans* including adhesive, acidogenicity, EPS, and biofilm formation.*Streptococcus mutans* effect and the inhibitory property may be due to suppression of the virulence factors of *S. mutans* including adhesive, acidogenicity, EPS, and biofilm formation.*S. mutans* was determined by the broth dilution method.

## 1. Introduction

Dental caries is a prevalent chronic oral infectious disease which is featured with progressive destruction of dental hard tissue, and as one of the most prevalent infectious diseases worldwide, dental caries endangers human health throughout the life cycle and nowadays we are even suffering an elevated risk for the incidence of dental caries [1, 2]. Vast studies on the etiology of caries reveal that multispecies microorganisms play an essential role in the occurrence and development of tooth decay, among which *S. mutans* is deemed as the main cariogenic bacteria species [1, 3–8]. To thrive in the oral microbiota, *S. mutans* managed to evolve with several cariogenic characteristics including the ability to adhere to tooth surface, to survive in low pH, and to produce acids and exopolysaccharides (EPS) [1, 9, 10], while the conversion of diet-derived carbohydrates into EPS can further induce the formation of biofilms [11]. In recent years, using antimicrobial agents as an adjuvant for oral mechanical therapy has received much attention and has been widely used in clinical practice, but long-term use of antibiotics will cause certain toxic and side effects, resulting in flora imbalance and antibiotic resistance [12]. Thus, it is important to explore new drugs that inhibit common oral pathogenic bacteria while not necessarily lead to medical tolerance.

To date, antibacterial peptides (AMPs) have attracted much attention as a promising alternative anti-infective for caries treatment [13–17]. AMP is a kind of small molecular polypeptide produced by the natural immune system, which widely exists in plants, insects, and mammals and has a broad-spectrum antimicrobial activity [18, 19]. AMPs have an inhibitory effect on multiple species of bacteria, fungus, and even viruses [20–24]. Moreover, AMPs are effective against both planktonic bacteria and bacterial biofilms [25–29]. Although the specific mechanism of AMPs varies based on the amino acid composition and physicochemical properties, positively charged residues contained in most of the AMPs allow them to interact with the negatively charged bacterial membranes [30–32] and then with membrane depolarization, membrane damage, pore formation, cell lysis, peptide internalization, and intracellular targets damage [30, 31]. These specific antimicrobial mechanisms of AMPs make them do not cause resistance easily. With the above mentioned properties, AMPs show great potential for clinical application, leading the research and medication of AMPs to raise increasing attention in fields of biopharmaceuticals [33]. However, most natural AMPs still have many disadvantages in their clinical application, including their large size, high cost and difficulty of production, and varying effective concentration against saliva dilution and degradation [34]. To improve these circumstances, taking natural AMPs as templates, many scholars have successfully designed and created many synthetic AMPs with promising antibacterial activity [35–37]. These years, Chen et al. [38] designed ZXR-2, Sullivan et al. [27] synthesized C16G2, and Zhang et al. [39] created DPS-PI, which were all synthetic AMPs that showed apparent antibacterial effect against the caries pathogenic bacteria, *S. mutans*. When compared with natural AMPs, synthetic AMPs can possess more efficient and broader-spectrum antibacterial activity and are not easy to produce resistance limitation, with lower cytotoxicity [34, 35, 40].

In recent years, many natural and synthetic AMPs have been confirmed to be inhibitory against cariogenic bacteria, defensins, Histatin 5, Human Lactoferrin [28], KSL [26], L-K6 [41], and C16G2 [42] included. And an increasing number of novel AMPs with kinds of properties are being discovered or synthesized.

GERM CLEAN is a new synthetic polypeptide. According to the manufacturer's instructions, it can effectively kill the pathogenic bacteria leading to oral mucositis, periodontitis, etc. As a novel drug product, there is no report on the antibacterial activity of GERM CLEAN on *S. mutans*. The aim of this study was to explore effects and the related mechanisms of GERM CLEAN on the major cariogenic bacteria, *S. mutans*, in the state of plankton and biofilm, so as to provide new perspective for the treatment of caries, as well as the experimental basis for further clinical promotion of this novel biological product.

## 2. Materials and Methods

### 2.1. Bacterial Strains and Growth Conditions

All chemicals and assay kits were purchased from Sigma-Aldrich (St. Louis, MO) unless otherwise stated. *S. mutans* UA159 was kindly donated by Dr. Justin Merrit from the University of Oklahoma Health Sciences Center and grown in brain–heart infusion broth (BHI; Oxiod, Basingstoke, UK) anaerobically (in an atmosphere consisting of 85% N_2_, 10% H_2_, and 5% CO_2_) at 37°C [43]. Overnight cultures of UA159 were diluted 20-fold in fresh BHI and grown to OD_600nm_ = 0.5 to generate mid-exponential phase bacteria. Mid-exponential phase bacteria cultures were further 20-fold diluted for *S. mutans* initial adhesion and biofilm formation using BHI medium supplemented with 1% (wt./vol) sucrose (BHIS).

### 2.2. The Filter Paper Disk Agar Diffusion Method

The antibacterial activity of GERM CLEAN (Shanxin, Chengdu, Sichuan, China) on *S. mutans* was preliminarily tested using the filter paper disc agar diffusion method introduced elsewhere with minor modification [44–47]. Briefly, 100 *μ*l of mid-exponential phase UA159 suspension was spread on fresh nutrient BHI agar medium plates and dried at room temperature for 5 min. The 5 mm sterile filter paper disks were impregnated with GERM CLEAN (the stock solution) for 5 s and then were applied to the surface of above BHI bacterial culture plates. Plates were then incubated for 24 h at 37°C. The antibacterial activity was evaluated by measuring the diameter of the inhibition zone.

The experiments were repeated three times independently.

### 2.3. Minimal Inhibitory Concentration (MIC) Determination

The broth microdilution method according to the previous introduction [44, 48–50] with some modifications was applied to determine the MIC value of GERM CLEAN towards *S. mutans*. Two-fold serial dilutions with BHI of GERM CLEAN were prepared in 96-well microtiter plates and the final mass fractions of the tested liquor were from 100% to 0.78%. First, 200 *μ*l of GERM CLEAN with a 100% mass fraction was added to the initial well. Next, 100 *μ*L of the BHI medium was added to other wells. Then, 100 *μ*l of 100% GERM CLEAN from the first well was added to the second well. After mixing, 100 *μ*l of this mixture was embedded into the following well. Similarly, the dilution procedure was continued to the 8th well. 10 *μ*L of the 1/20th mid-exponential phase bacterial suspension with a standard concentration of 0.5 (OD_600nm_) was added to each well. The BHI medium was used as a negative control. The 96-well microtiter plate was then incubated for 24 h at 37°C under the anaerobic conditions mentioned above. The MIC was defined as the lowest concentration of GERM CLEAN with no visible bacteria existing and the well looked clear and transparent.

The experiments were repeated three times independently.

### 2.4. Growth Curve Assay

We diluted the *S. mutans* of mid-exponential phase with BHI broth to obtain the starting optical density at 600 nm of 0.05. Then, we added GERM CLEAN into the 96-well microtiter plate filled with *S. mutans* culture to a final concentration of 1/2MIC. BHI medium acted as a negative control. The growth of 200 *μ*l cultures in a 96-well microtiter plate was measured. The 96-well microtiter plate was incubated at 37°C anaerobically 24 h and optical density at 600 nm was determined using a microplate spectrophotometer (Multiskan GO; Thermo Scientific, Waltham, MA) every hour throughout 24 h of incubation.

The experiment was repeated three times independently.

### 2.5. Initial Adhesive Assay

The initial adhesive assay was performed in 48-well microtiter plates. Mid-exponential *S. mutans* was diluted with BHIS as described above. Then, we added GERM CLEAN into the 48-well microtiter plates filled with *S. mutans* culture to final concentrations of 1/2MIC. BHIS medium acted as a negative control. The microtiter plates were incubated anaerobically at 37°C for 1 h, 2 h, and 4 h, respectively. After incubation, we removed the suspension and washed the wells twice with PBS to obtain the adherent cells and then added 500 *μ*l sterile BHIS broth to resuspend the adherent cells. Amount of adherent bacteria was determined by measuring optical density at 600 nm and the difference of OD_600nm_ between treated and control groups was compared. To further evaluate the effect of GERM CLEAN at the concentration of 1/2MIC on the adherence of *S. mutans*, we calculated the antiadherence percentage.

The experiment was repeated three times independently.

The initial adherence [51, 52]: OD_600nm_ of assay group compared with OD_600nm_ of control group.

Antiadherence percentage [49, 53] = (OD_600nm_ of control group − OD_600nm_ of assay group)/OD_600nm_ of control group × 100%.

### 2.6. Biofilm Formation Assay

The effect of GERM CLEAN on *S. mutans* biofilm formation was explored using a quantitative crystal violet assay described elsewhere [54, 55] with some modifications. Briefly, mid-exponential *S. mutans* was diluted with BHIS broth as described above. Then, we added GERM CLEAN into the 96-well microtiter plate filled with *S. mutans* culture to final concentrations of 1/2MIC. BHIS medium acted as a negative control. After anaerobic incubation (24 h, 37°C), culture supernatants, and planktonic cells were removed, and the biofilm in each well was washed with PBS to remove the remaining unattached cells. The biofilms were then fixed with methanol for 15 min and stained with 0.1% (wt./vol) crystal violet for 15 min, sequentially. After staining, the biofilm was rinsed twice with distilled water to remove excess CV, and then the dye bound to the cells was resolubilized with 33% (vol/vol) glacial acetic acid for 20–30 min at room temperature. Biofilm formation was then quantified by measuring the optical density of the suspension at 600 nm by a microplate reader (Gene, Hong Kong, China).

The experiment was repeated three times independently.

### 2.7. Water-Insoluble EPS Measurement

The anthrone method [43, 56] was used to examine the effect of GERM CLEAN on production of water-insoluble EPS by *S. mutans* with some modifications. Briefly, biofilms were collected by sonication/vortexing in PBS buffer. Then, the precipitate was obtained by centrifugation (4000 rpm, 10 min, 4°C), washed twice with sterile water, and resuspended in 1 ml of 0.4 M NaOH. Water-insoluble polysaccharides were extracted under agitation for 2 h at 37°C. After centrifugation (4000 rpm, 10 min, 4°C), we added three volumes of 0.2% anthrone-sulfuric acid reagent to each supernatant sample at 95°C for 6 min. The OD_625nm_ was monitored with a microplate reader.

The experiment was repeated three times independently.

### 2.8. Scanning Electron Microscope (SEM) Examination

The biofilms were produced in a 24-well plate with sterilized glass slides at the bottom of wells. The biofilms were formed as described above. The specimens were rinsed with PBS three times and then fixed with 2.5% glutaraldehyde overnight at 4°C. Following initial fixation, the specimens were washed with PBS and then serial dehydrated with ethanol (30%, 50%, 70%, 80%, 85%, 90%, 95%, and 100%) for 30 min each time; finally, the biofilms were dried and observed at magnifications of 5,000*x* and 20,000*x* by SEM imaging (FEI, Hillsboro, USA).

### 2.9. Confocal Laser Scanning Microscope (CLSM) Examination

The biofilm specimens were formed on sterile glass coverslips put at the bottom of 24-well microtiter plates as described above. The specimens were rinsed with PBS three times and CLSM imaging was used for observation of the live/dead staining of *S. mutans* biofilms. Briefly, biofilms were stained using the LIVE/DEAD1 BacLight™ Bacterial Viability Kit (L-7012, Molecular Probes™, Invitrogen, Carlsbad, CA, USA) containing two component dyes (SYTO 9 and propidium iodide) following the manufacturer's instruction. The labeled biofilms were imaged with a confocal laser scanning microscope (DMIRE2, Leica, Wetzlar, Germany) equipped with a 60 × oil immersion objective lens. The image channels were set according to the manufacturer. The excitation maxima for these dyes were 480/500 nm for the live cell stain SYTO 9 and 490/635 nm for the dead cell stain propidium iodide. Each biofilm was scanned at five randomly selected positions.

### 2.10. Glycolytic Rate Assay

The effect of GERM CLEAN on *S. mutans* glycolysis was measured by pH drop assay as described elsewhere with some modifications [54, 57, 58]. Briefly, *S. mutans* was harvested at mid-logarithmic phase (10,000 g, 10 min, 4°C), washed twice with salt solution (50 mM KCl + 1 mM MgCl_2_, PH = 7.2), and resuspended in the same salt solution containing GERM CLEAN at the concentration of 1/2MIC. Samples resuspended in BHIS served as a negative control. Glucose was added to obtain a final concentration of 1% (wt./vol) to trigger glycolysis, and the decrease in pH of the bacterial suspensions was evaluated over a period of 75 min using a glass electrode (Thermo Scientific, Waltham, MA).

The experiment was repeated three times independently.

### 2.11. Quantitative Real-Time PCR (qRT-PCR)

QRT-PCR was used to examine the effect of GERM CLEAN on expression levels of *S. mutans* virulence trait related genes including *gtfB, gtfC, gtfD*, and *ldh*, and *gyrA* was used as the internal control for quantification [56, 59–61]. Mid-exponential phase *S. mutans* was 20-fold diluted in BHI broth, and then the bacteria culture was grown in the BHI broth with 1/2MIC level of GERM CLEAN, while BHI broth with no GERM CLEAN acted as a control.

The RNA isolation and purification procedures were conducted according to Xu et al.'s protocol [62]. RNA reverse transcription was performed with a PrimeScript™ RT reagent kit (Takara Biotechnology, Japan) to synthesize first-strand cDNAs. Specific primers for target genes were designed according to other studies [43, 56, 61] and listed in Table 1. Each qRT-PCR reaction mixture contained SYBR® Premix Ex Taq™ II (RR820A; Takara Bio), cDNA samples (1 *μ*l), and forward and reverse gene-specific primers (10 *μ*M/l, 0.5 *μ*l each). The qPCR was performed on the CFX96 Real-Time System (C1000™ Thermal Cycler; Bio-Rad, Hercules, CA) applying the thermal cycling conditions described in Ming-Yun et al.'s protocol [63]. Relative expression fold changes of tested genes were calculated using the 2^−ΔΔCt^ method, and expression level of *gyrA* rRNA gene was used to normalize the expression level of different genes.

The experiments were repeated three times independently.

### 2.12. Statistical Analysis

Differences between the experimental group and the untreated control group were compared using the *t*-test after a homogeneity test of variance with Levene's test except for evaluations of the inhibition zone and antiadherence percentage, where the Wilcoxon Signed Ranks Test was used for the former and the Student-Newman-Keuls Test was used for the latter after the aforementioned homogeneity test. Statistical analysis was performed using SPSS software (Version 20.0; IBM Corp, Armonk, USA) at a significance level of 0.05, and then all of our figures were obtained using the Graphpad Prism7 software (version 7.00 for Windows; GraphPad Prism, Inc, La Jolla, USA) according to the analysis results.

## 3. Results

### 3.1. Growth Inhibition Zone Diameter Determination

The filter paper disks saturated with the stock GERM CLEAN solution could form inhibition zones on the bacterial culture plates, and diameters of the inhibition zones were 10.18 ± 1.744 mm (>7 mm, *p* < 0.05), which preliminarily indicated the antibacterial effect of GERM CLEAN on *S. mutans*.

### 3.2. Minimal Inhibitory Concentration (MIC) Determination

After 24 h incubation, the MIC value of GERM CLEAN against *S. mutans* obtained by the broth microdilution method was 100% mass fraction, which was the stock solution.

### 3.3. Growth Curve Assay

**Figure 1:**
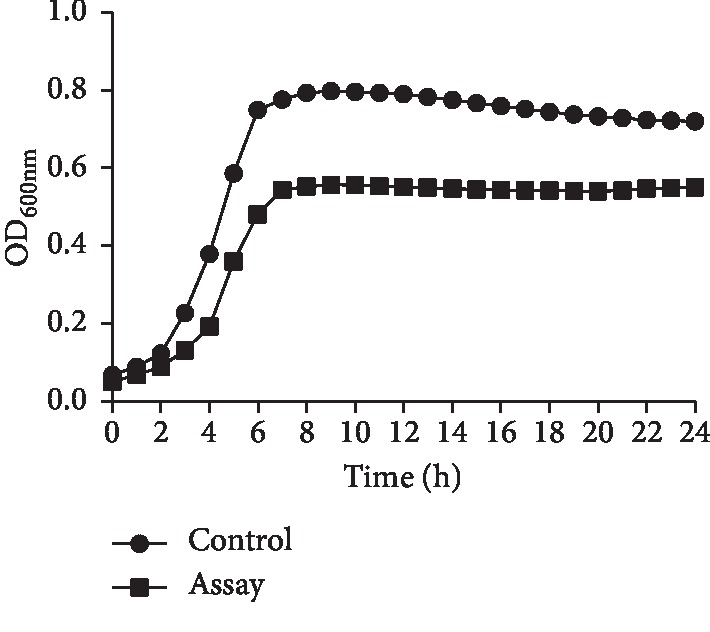
Effect of GERM CLEAN at 1/2MIC on the growth curve of *S. mutans*.

We evaluated the effect of GERM CLEAN at 1/2MIC level on the basic viability of *S. mutans* by growth curve. As shown in Figure 1, the growth curves of GERM CLEAN-treated and GERM CLEAN-untreated *S. mutans* exhibited significant differences. It was observed that *S. mutans* treated with GERM CLEAN at 1/2MIC exhibited an extended lag phase. *S. mutans* of control group entered the logarithmic phase after 3 h and showed rapid growth till 10 h with a higher-end OD_600nm_ of 0.8. However, when treated with GERM CLEAN at 1/2MIC, *S. mutans* grew a little more slowly with the lag phase extending to 4 h and lower final OD_600nm_ of 0.55. GERM CLEAN obviously decrease the number of final bacterial concentration, but it did not dramatically delay the progress of bacteria to logarithmic growth.

### 3.4. GERM CLEAN Inhibits the Initial Adherence of *S. mutans In Vitro*

**Figure 2:**
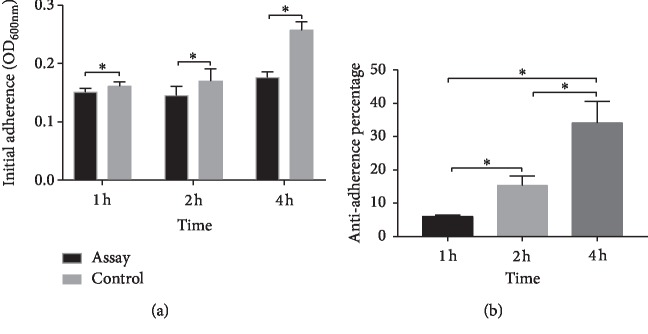
The anti-adherence effect of 1/2MIC GERM CLEAN on *S. mutans*. (a) The OD_600nm_ of adherent bacteria treated with 1/2MIC level of GERM CLEAN or BHIS control; (b) the antiadherence percentage of the initial adherence stage (1 h, 2 h, and 4 h) calculated by (OD600 nm of control group − OD_600nm_ of assay group)/OD600 nm of control group. ^*∗*^*p* < 0.05.

The inhibition capacity of GERM CLEAN at 1/2MIC on *S. mutans* biofilm formation was analyzed by calculating the antiadhesion percentage. As shown in Figure 2(a), GERM CLEAN reduced *S. mutans* adhesion (*p* < 0.05) after 1 h, 2 h, and 4 h of inoculating for biofilm formation.  Figure 2(b) showed that GERM CLEAN effectively reduced the adherence of *S. mutans* in a time-dependent manner. Specifically, during the first 4 h after inoculation, the anti-adherence percentage increased along with the time from 6.0% at 1 h to 15.2% at 2 h and ended up with 34.1% at 4 h.

### 3.5. GERM CLEAN Inhibits Water-Insoluble EPS Synthesis and Biofilm Formation of *S. mutans In Vitro*

**Figure 3:**
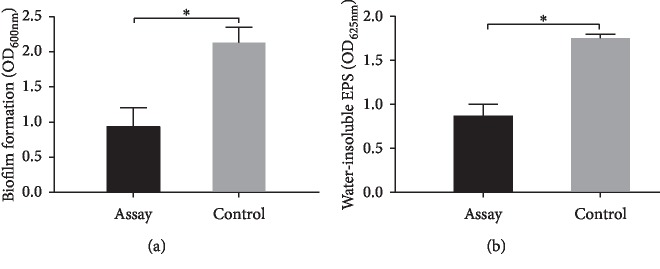
Effect of 1/2MIC GERM CLEAN on biofilm formation and water-insoluble EPS of *S. mutans.* (a) Quantitative data of the biofilm formation measured by crystal violet dye; (b) the water-insoluble EPS measured by the anthrone method. ^*∗*^*p* < 0.05.

GERM CLEAN at 1/2MIC level impaired *S. mutans* biofilm formation and disrupted the ability of *S. mutans* to synthesize water-insoluble EPS (Figure 3). Being treated with GERM CLEAN reduced (*p* < 0.05) up to 56.24% of biofilm formation when compared with the control group (Figure 3(a)). The water-insoluble EPS in the treated group decreased 51.76% (*p* < 0.05) compared with the control group (Figure 3(b)).

### 3.6. Scanning Electron Microscopy Examination

**Figure 4:**
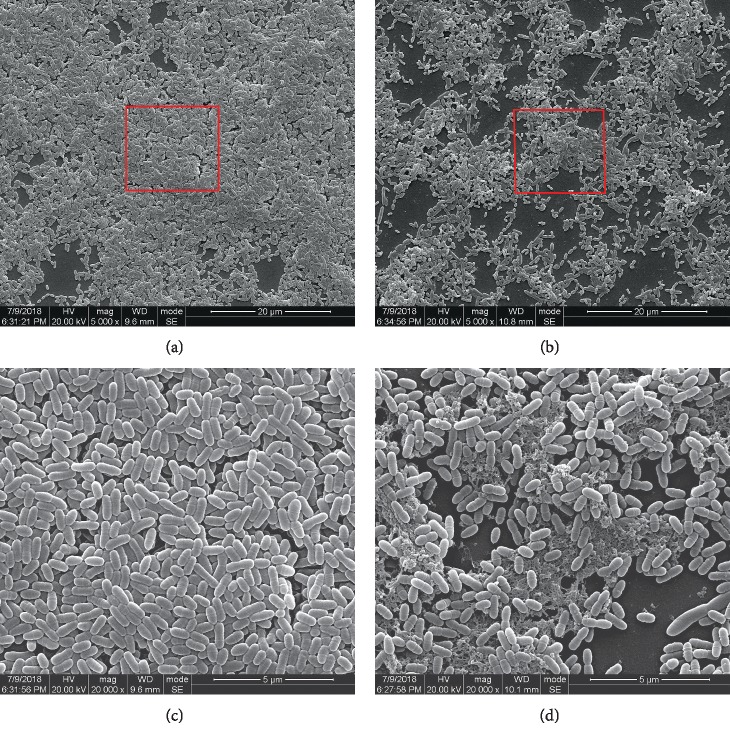
Scanning electron microscopy observation of *S. mutans* biofilm formed after 24 h of incubation. (a) and (c) were taken from the untreated control group, while (b) and (d) represented the group treated with 1/2 MIC concentration of GERM CLEAN. (a) and (b) 5000*x*; (c) and (d) partially magnified (20000*x*) from the red circles in (a) and (b), respectively.

SEM imaging depicted the impact of GERM CLEAN on *S. mutans* glucan production and biofilm structure (Figure 4). The BHIS treated control group displayed smooth cell surface with neither apparent cell lysis nor cellular debris, while it showed evident aggregation of cells with the formation of chains immersed into the EPS pool. In accordance with the results of our initial adhesion, anthrone, and biofilm quantifying assays, the GERM CLEAN-treated samples displayed rough, shrunken, distorted, and collapsed cells and obvious membrane rupture with significant dispersion of the cells, which also suggested the reduced production of EPS. And the cells in the GERM CLEAN-treated samples showed irregular shapes and obviously various sizes, with the occurrence of polarity.

### 3.7. Confocal Laser Scanning Microscopy (CLSM) Examination

**Figure 5:**
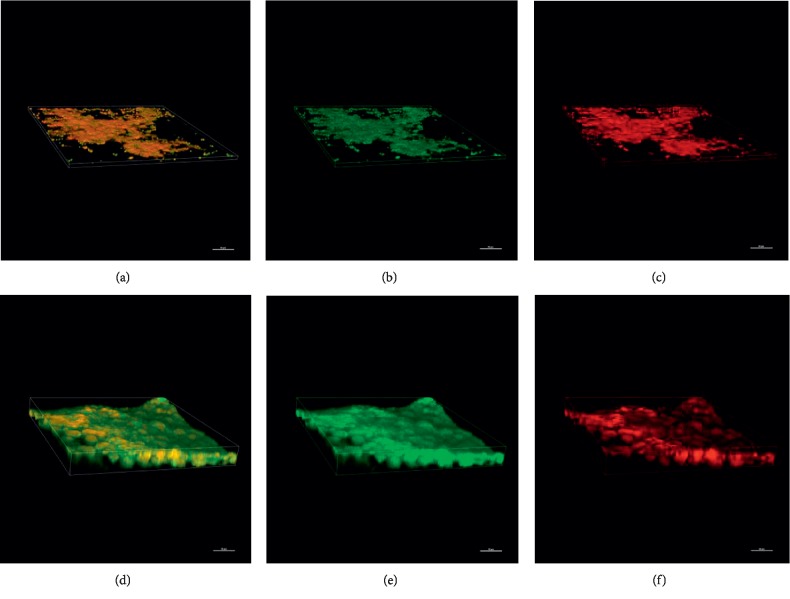
CLSM images of *S. mutans* biofilm formed in the presence and absence of the sub-MIC (1/2MIC) level of GERM CLEAN after 24 h of incubation. (a), (b), and (c) were from the treated group; (d), (e), and (f) were from the control group; (a) and (d) show the whole biofilm images; live bacteria, stained green (b, e); dead cells, stained red (c, f).

The effect of GERM CLEAN at 1/2MIC concentration on the biofilm architecture of *S. mutans* was also analyzed by CLSM. As shown in Figure 5, GERM CLEAN showed a clear antibacterial effect that was mainly constituted of destruction of biofilms and reduction of living cells. In the control group, *S. mutans* biofilms were compact and most of the bacteria were viable. However, when treated with GERM CLEAN, *S. mutans* formed unconsolidated biofilms with an apparent scattering of cells, where viable cells were diminished, while cell deaths increased.

### 3.8. GERM CLEAN Inhibits the Acidogenicity of *S. mutans In Vitro*

**Figure 6:**
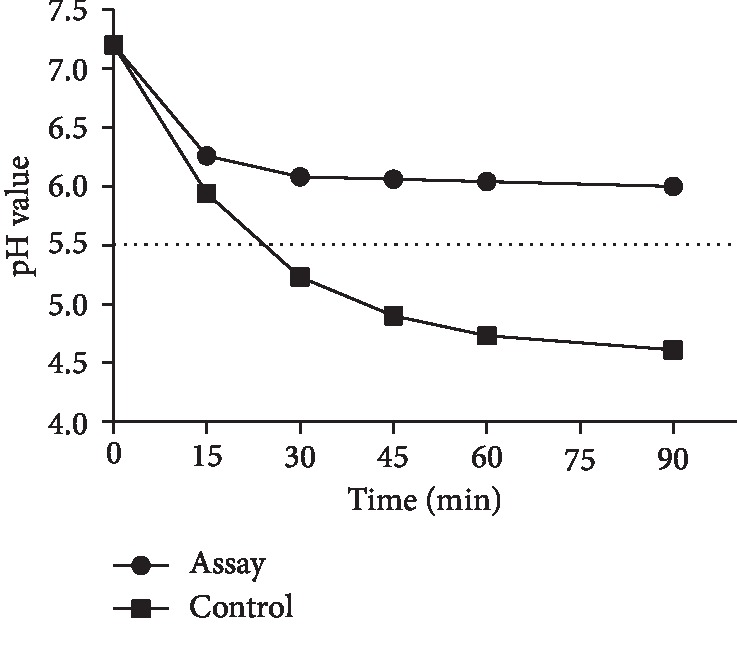
Effect of 1/2MIC concentration of GERM CLEAN on *S. mutans* acid production measured by glycolytic pH drop assay. The horizontal dotted line represents the critical pH value (pH 5.5) to *S. mutans*.

Results of the pH drop assay suggested that GERM CLEAN at the concentration of 1/2MIC level repressed *S. mutans* acidogenicity. As shown in Figure 6, the pH drop recorded in the first 15 min of incubation (known as initial pH drop) was observed maximum in both control and the GERM CLEAN-treated groups; in the control group, the pH of the bacterial suspension decreased quickly from 7.2 to 5.94 and then slowly ended up with 4.65 after 90 min of incubation, whereas the pH value of GREM CLEAN-treated group decreased quickly from 7.2 to 6.26 and slowly showed a higher final pH of 5.96 (*p* < 0.05).

### 3.9. GERM CLEAN Inhibits Expression of Virulence Genes of *S. mutans In Vitro*

**Figure 7:**
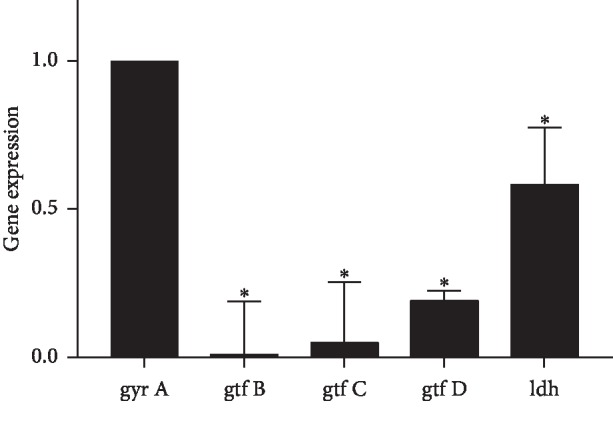
Expression of various virulence genes of *S. mutans* in response to the treatment with GERM CLEAN. Gene expression was quantified by real-time PCR, with *gyrA* rRNA as an internal control. ^*∗*^*p* < 0.05.

The expression fold changes of *ldh, gtfB, gtfC,* and *gtfD* in *S. mutans* treated with GERM CLEAN at 1/2MIC level were shown in Figure 7. When compared with gene expressions in the control group, all those of these tested genes were downregulated after treating with GERM CLEAN (*p* < 0.05), especially for that of the *gtfB* gene, whose expression level was decreased by nearly 100-fold. And the expression levels of *gtfC, gtfD*, and *ldh* were downregulated by about 20-fold, 5-fold, and 2-fold, respectively, after treated with GERM CLEAN at 1/2MIC level.

## 4. Discussion

In this study, we found that a newly marketed oral spray GERM CLEAN impaired the growth, adherence, EPS synthesis, biofilm formation, and acid production of *S. mutans* through *in vitro S. mutans* virulence-related phenotypic assays. Moreover, the qRT-PCR result explained that GERM CLEAN impaired virulence of *S. mutans* through downregulating expression of EPS- and acid-production related genes.

The antibacterial activity of GERM CLEAN was preliminarily verified through the antibacterial ring test, but the diameter of inhibition zones formed by GERM CLEAN was not very stable, which varied from 8.4 mm to 12 mm according to our repeated tests, and we suspected that it might be related to the variety of the peptide stability [64–69], and this hypothesis needs further confirmation. The MIC of GERM CLEAN turned out to be 100% mass fraction, which was unexpected, but on the other hand, this also indicated the mild and biocompatible properties of GERM CLEAN.

As a primary etiology of dental caries, *S. mutans* poses a strong adhesive ability to attach to the tooth surface, which is the decisive initial step in colonization, biofilm formation, and caries development [70–72]. Water-insoluble EPS in the matrix also plays a critical role in *S. mutans* carcinogenicity [73, 74]. EPS can promote the aggregation of bacteria to form a biofilm, thus displaying cariogenic properties [4, 10, 72]. Biofilm is responsible for caries and EPS consolidates it, which increases the resistance of antibacterial reagents [75, 76]. Glucosyltransferases (*Gtfs*) secreted by *S. mutans* are the key enzymes mediating glucan synthesis, which impair the following adherence and biofilm formation. *GtfB, GtfC*, and *GtfD* are encoded by *gtfb, gtfc*, and *gtfd* genes, respectively. *GtfB* makes primarily water-insoluble glucans, *GtfC* appears to synthesize both soluble and insoluble glucans, with water-insoluble glucans predominating, and GtfD mainly makes water-soluble glucans [77–84]. Water-insoluble glucans synthesized by *GtfB* and *GtfC* form the main scaffold of the EPS matrix and provide adhesive sites for *S. mutans* to a tooth surface as well as to other microbes [80–82]. Previous studies demonstrated that suppressed expression of *gtfBC* genes in *S. mutans* could ultimately inhibit the biofilm formation because of the reduction of EPS and adherent ability [79, 83, 85–87]. Nowadays, study groups developed an increasing number of novel synthetic AMPs with confirmed antibacterial potential. Wang et al. [88] synthesized TVH19 and testified its effect on inhibiting the biofilm formation and destroying the biofilm structure of *S. mutans*. Min et al. [13] synthesized CLP-4 and demonstrated that CLP-4 could kill *S. mutans* cells, inhibit biofilm formation, and eradicate preformed biofilms. Jannadi et al. [89] synthesized Pep19-2.5 and Pep19-4LF and assessed that they inhibited *S. mutans* growth and biofilm formation. Similarly, Zhang et al. [39] designed DPS-PI, Liang et al. [90] designed LR-10, Da Silva et al. [91] designed [W7]KR12-KAEK, and all of their antibacterial activities were evaluated by assessing the inhibition of *S. mutans* growth and biofilm biomass, furthermore assessing the destruction to biofilm morphology and the damage to the bacterial surface via scanning electron microscopy. In this current study, GERM CLEAN showed capabilities on reducing the initial adherence and disrupting the biofilm formation of *S. mutans*. Results in the anthrone experiment revealed that GERM CLEAN reduced EPS synthesis, and downregulated EPS-production related gene (*gtfB, gtfC*) expression levels further conformed the inhibition effect of GERM CLEAN on the EPS-production ability of *S. mutans,* which could mediate ineffective adhesion and biofilm formation. According to the negative effects of GERM CLEAN on *S. mutans* adhesion, biofilm formation, and EPS production, we speculate that GERM CLEAN could disrupt bacterial aggregation on the tooth surface and thereby the biofilm formation, thus playing a promising role in the prevention and treatment of caries. The CLSM and SEM demonstrated GERM CLEAN disrupted biofilm formation by reducing the composition of live bacteria and distorting the biofilm structure. And we speculated that it was connected with the decrease of EPS synthesis, which was verified in the anthrone assay. And the SEM showed that GERM CLEAN could cause apparent cell lysis, cellular debris, pore formation, and obvious membrane rupture, which indicated that the possible antibacterial mechanism of our AMP may be the commonly accepted electrostatic interactions.

Acid production is another noteworthy pathogenic feature of *S. mutans*. Lactate dehydrogenase (*LDH*) encoded by *ldh* gene is one of the most important enzymes in acid production, which acts as a key virulence of *S. mutans,* and *ldh*^−^ deficient mutant of *S. mutans* had low acidogenicity and reduced cariogenic potential [92–98]. In a previous study, Wang [43] synthesized GH12 and performed glycolysis pH drop assay and qRT-PCR to test its effect on acid production of *S. mutans*. With similar methods in this research, our data from the glycolysis pH drop assay suggested the impairment effect of GERM CLEAN on acidogenicity of *S. mutans* and this result was consistent with qRT-PCR data which showed that GERM CLEAN could downregulate expression of acid-production related gene *ldh*. GERM CLEAN clearly repressed acid generation of *S. mutans*, implying its prevention effect on *S. mutans* derived tooth erosion and demineralization, which consequentially inhibits *S. mutans* carcinogenicity. On the other hand, lower final pH value was related to the ability of stronger acid tolerance [73] to some extent, so the higher final PH value after being treated with GERM CLEAN at 1/2MIC indicated the disruption of aciduric potential, which further impaired the cariogenic ability of *S. mutans*.

The overall effect of GERM CLEAN is evidently anticariogenic as shown by *in vitro* studies. All results concluded that GERM CLEAN at 1/2MIC suppressed the cariogenic pathways of *S. mutans*. But bacteriostasis on *S. mutans* of GERM CLEAN in poor stability is worth further thinking, and extraordinary comparison found that the antibacterial effect of GERM CLEAN is poorer than clinical commonly used chlorhexidine (CHX) which has a definite effect. CHX, widely used as a mouthwash, is one of the most commonly prescribed antiseptic agents in dentistry due to broad-spectrum antimicrobial activity [99–101]. It adheres to tooth and mucosal surfaces and presents a high residence time and it is considered a gold standard for dental caries and periodontitis control [99, 100, 102–106]. However, CHX has some side effects which limit our common use, including tooth discoloration, impaired sense of taste, mucosal desquamation, and irritation of host tissues [103, 107–109]. What's more, CHX mouthwash needs to be gargled and its use is restricted by our environment and location. To our delight, GERM CLEAN, existing as an AMP spray which has a clear antibacterial effect, is easy to be carried. Moreover, it is mild, colorless, and tasteless, with good user experience, which overcomes the CHX's shortcomings to some degree.

So far, scholars have confirmed the antibacterial effect of many natural and synthetic AMPs, including Bac8c, Aedesin, decapeptide, XLAsp-P1, LL-37 and variant, Pep-7, and HBD3- C15 [35, 87, 110–116], which provided us with the study methods of this new AMP GERM CLEAN. What's more exciting, Wang et al. [43, 117] in our hospital successfully synthesized three new AMPs (GH8, GH12, and GH16) and further studied the bacteriostatic properties and mechanisms. Although reports about GERN CLEAN at home and abroad are limited before, these previous studies on other AMPs provide a classic and mature experimental scheme for the study of *in vitro* antibacterial effect and mechanisms of this new AMP for us.

However, we need to take the limitations of such *in vitro* assays into consideration. There are more than 700 species of bacteria residing on the teeth surfaces and oral soft tissues [102, 118]. Meanwhile, dental plaque is a multispecies biofilm [75, 102, 119], which could cause a variety of oral diseases including dental caries, pulpitis, gingivitis, and periodontitis [102, 118, 120, 121]. Therefore, in our further study, we need to examine the effect of GERM CLEAN on plaque biofilm containing multiple species of bacteria. Besides, considering the complicated bacteria-host interactions, *in vivo* animal models may help us observe the effect of GERM CLEAN more intuitively.

In conclusion, GERM CLEAN at 1/2MIC could reduce the acidogenicity, EPS synthesis, adherent ability, and biofilm formation of *S. mutans* through downregulating the expression levels of *gtfb, gtfc, gtfd*, and *ldh* genes. According to the manufactures' instruction, GERM CLEAN is an orally and topically administrated anti-infectious agent, and based on the finding of our present *in vitro* study, we assumed that this spray could exert antibacterial effect on the main cariogenic bacteria *S. mutans*, which suggested that this product might be useful in terms of caries prevention and treatment, and it should be especially recommended to apply this novel spray to patients with a predicted high-risk of caries. However, considering the diversity and complexity of human dental plaque, further studies with animal and clinical patient trials are still needed for figuring out the best fit indications and the actual performance of GREM CLEAN *in vivo*.

## Figures and Tables

**Table 1:** Specific primers of quantitative real-time PCR.

Primers	Sequence	References
*gyrA*-F	5′-ATTGTTGCTCGGGCTCTTCCAG-3′	[56, 61]
*gyrA*-R	5′-ATGCGGCTTGTCAGGAGTAACC-3′	
*gtfB*-F	5′-CACTATCGGCGGTTACGAAT-3′	[43]
*gtfB*-R	5′-CAATTTGGAGCAAGTCAGCA-3′	
*gtfC*-F	5′-GATGCTGCAAACTTCGAACA-3′	[43]
*gtfC*-R	5′-TATTGACGCTGCGTTTCTTG-3′	
*gtfD*-F	5′-TTGACGGTGTTCGTGTTGAT-3′	[43]
*gtfD*-R	5′-AAAGCGATAGGCGCAGTTTA-3′	
*ldh*-F	5′-AAAAACCAGGCGAAACTCGC-3′	[43]
*ldh*-R	5′-CTGAACGCGCATCAACATCA-3′	

## Data Availability

The data used to support the findings of this study are available from the corresponding author upon request.
